# *Plasmodium vivax *circumsporozoite genotypes: a limited variation or new subspecies with major biological consequences?

**DOI:** 10.1186/1475-2875-9-178

**Published:** 2010-06-23

**Authors:** Wanessa C Souza-Neiras, Luciane M Storti-Melo, Gustavo C Cassiano, Vanja SCA Couto, Álvaro ARA Couto, Irene S Soares, Luzia H Carvalho, Maristela G Cunha, Marinete M Póvoa, Socrates Herrera, Myriam A Herrera, Andrea RB Rossit, Claudia MA Carareto, Ricardo LD Machado

**Affiliations:** 1 Departamento de Biologia, Universidade Estadual Paulista "Júlio Mesquita Filho", São José do Rio Preto, São Paulo State, Brazil; 2 Faculdade SEAMA, Macapá, Amapá State, Brazil; 3 Departamento de Análises Clínicas e Toxicológicas, Faculdade de Ciências Farmacêuticas, Universidade de São Paulo, São Paulo State, Brazil; 4 Centro de Pesquisas René Rachou, Fundação Oswaldo Cruz, Belo Horizonte, Minas Gerais State, Brazil; 5 Universidade Federal do Pará, Instituto de Ciências Biológicas, Belém, Pará State, Brazil; 6 Instituto Evandro Chagas, MS/SVS, Ananindeua, Pará State, Brazil; 7 Instituto de Imunologia, Universidade Del Valle, Cali, Colômbia; 8 Centro de Investigação de Microrganismos, Departamento de Doenças Dermatológicas, Infecciosas e Parasitárias, Faculdade de Medicina de São José do Rio Preto, São Paulo State, Brazil

## Abstract

**Background:**

*Plasmodium vivax *circumsporozoite variants have been identified in several geographical areas. The real implication of the genetic variation in this region of the *P. vivax *genome has been questioned for a long time. Although previous studies have observed significant association between VK210 and the Duffy blood group, we present here that evidences of this variation are limited to the CSP central portion.

**Methods:**

The phylogenetic analyses were accomplished starting from the amplification of conserved domains of *18 SSU RNAr *and *Cyt B*. The antibodies responses against the CSP peptides, MSP-1, AMA-1 and DBP were detected by ELISA, in plasma samples of individuals infected with two *P. vivax CS *genotypes: VK210 and *P. vivax*-like.

**Results:**

These analyses of the two markers demonstrate high similarity among the *P. vivax CS *genotypes and surprisingly showed diversity equal to zero between VK210 and *P. vivax*-like, positioning these *CS *genotypes in the same clade. A high frequency IgG antibody against the N- and C-terminal regions of the *P. vivax *CSP was found as compared to the immune response to the R- and V- repetitive regions (*p *= 0.0005, Fisher's Exact test). This difference was more pronounced when the *P. vivax*-like variant was present in the infection (*p *= 0.003, Fisher's Exact test). A high frequency of antibody response against MSP-1 and AMA-1 peptides was observed for all *P. vivax CS *genotypes in comparison to the same frequency for DBP.

**Conclusions:**

This results target that the differences among the *P. vivax CS *variants are restrict to the central repeated region of the protein, mostly nucleotide variation with important serological consequences.

## Background

The circumsporozoite surface protein (CSP) is the most abundant polypeptide present in the sporozoite covering. This protein is involved in the motility and invasion of the sporozoite during its entrance in the hepatocyte [1,2].

Some years ago, CSP was studied as the main goal for anti-malarial vaccine development; however the existence of variations in the repetitive sequence of its central portion has been hindering these studies. *Plasmodium vivax *CSP sequences analyses revealed that parasites have repeats belonging to one of two types of nonapeptide repeat units, GDRA(A/D)GQPA or ANGA(G/D)(N/D)QPG, named VK210 or VK247 respectively [3,4]. In 1993, a new human malaria parasite from a *P. vivax*-infected person was identified by Qari *et al *[5], who named it *P. vivax*-like. The CSP sequence of *P. vivax*-like has an 11-mer repeat sequence, APGANQ(E/G)GGAA, and is different to the two previously described variants [5,6].

All *P. vivax CS *genotypes have a worldwide distribution and have been identified for several authors [7-17]. In Brazil, the occurrence of the three genotypes in pure and mixed infections was described [11,17]. Seroreactivity tests have identified the presence of three variant genotypes in samples from the State of São Paulo [10,16] and in indigenous populations [8,9] and other communities of the Amazon region [13]. Studies have also reported differences in the infectivity of anophelines to the variant genotypes, indicating that *Anopheles darlingi *and *Anopheles pseudopunctipennis *were more susceptible to the infection by VK210 [18,19]. These findings could be a consequence of differences in the emergence of this genotype in specific geographical regions or suggest that the VK210 genotype is the best-adapted variant in the world [11].

The successful of the vaccine against malaria can be related to the immunological intervention in the development of the parasite in the human host or mosquito vector. To improve the health and quality of more than one billion people around the world, several efforts have been addressed for the identification and antigenic characterization of different *P. vivax *antigens, among these the preerythrocytic antigens such as circumsporozoite protein (CSP) [20], the blood-stage proteins as merozoite surface protein 1 (MSP-1) [21,22], apical membrane antigen 1 (AMA-1) [22,23], and the Duffy binding protein (DBP), an merozoite antigen that interacts with the Duffy blood group in the host cells surface [22,24]. Currently, several authors have considered the CSP of *P. vivax *as the major target for the development of recombinant malaria vaccines, since the synthetic peptides starting from this protein induce a high and specific humoral response as the induced by natural exposure of humans to malaria [25-31]. Moreover, starting from the description of the *P. vivax CS *genotypes, VK210, VK247 and *P. vivax*-like, several studies proposed the existence of differences among those that seem to go besides variations in the repetitive portion of the protein, as geographical distribution, transmission intensity, vectorial competence, immune and treatment responses and drug resistance [11,18,19,32-34].

Many studies are being conducted to better understand the age and origin of the *P. vivax *as a human parasite [35,36]. Low microsatellite and tandem repeat variability indicate that *P. vivax *infected humans recently (10,000 years ago) [37]. Indeed, a different study based on polymorphisms of two nuclear and one mitochondrial gene places this parasite origin between 45,000 and 81,000 years ago [35]. In addition, *P. vivax *seems to be related to the clade of parasites found in Asian cercopithecines, indicating its origin in Asia via a host-switch from parasites found in non-human primates, such as macaques [35,36,38]. Little it is known about the characterization of the *P. vivax *variants, since analyses of the non-repetitive portion of *CS *gene showed that these genotypes belong to a same clade, including several types of primate *Plasmodium *species [35,39]. Nevertheless, the important question remains whether the *P. vivax CS *repeated region is a limited, mostly simple base variation [40] or if these variants represent the existence of a new species or subspecies of *Plasmodium *causing human malaria, with major biological consequences [6].

Here, this work contributes to the understanding of the implication of the central repetitive region variation of the *CS *in the *P. vivax *genome by phylogenetic tools and to the evaluation of the humoral immune response against different parasite antigens.

## Methods

### Subjects

After given written informed consent, peripheral blood samples were drawn from malaria patients living in four Brazilian Amazon endemic areas (Macapá, Amapá State; Novo Repartimento, Pará State; Porto Velho, Rondônia State and Plácido de Castro, Acre State). All individuals enrolled in this study complied with the following criteria: they sought medical assistance for clinical malaria symptoms, were over 18 years old and had a positive malaria diagnosis by thick blood film for *P. vivax*. The genomic DNA was extracted by the phenol-chloroform method [41] or using a commercially available kit (Easy-DNA™, Invitrogen, USA), and a semi-nested PCR was performed using *P. vivax*-specific small-subunit (SSU) rDNA primers to confirm the *Plasmodium *diagnosis [42]. The *P. vivax CS *genotypes were determined as described by Alves *et al *[43].

### Molecular analyses

For the phylogenetic reconstruction, a subset of the *P. vivax *field samples was used and data for non-human *Plasmodium *spp. samples were obtained from GenBank. Natural hosts type, geographic origins and GenBank accession numbers of the out groups are described in additional file 1: Hosts type, geographic origins and GenBank accession numbers of the out groups. All amplification reactions were performed in a thermocycler (DNA MasterCycler; Eppendorf, USA). The PCR-amplified products were purified by using GFX (GE Healthcare, United Kingdom) and EXOSAP (USB, USA) PCR purification kits, according to the manufacturer's protocol. DNA sequencing was performed using the Big Dye™ Terminator V3.1 Cycle Sequencing kit on ABI 3100 Genetic Analyzer (Applied Biosystems, USA).

### Amplification of the molecular markers

#### *18 small sub unit ribosomal RNA (SSU rRNA) *gene analyses

The amplification of a target area between variable regions 7 and 8 of the *18 SSU rRNA *gene from *P. vivax *was designed as described by Santos-Ciminera *et al *[44]. PCR was performed using the primer pairs VAR1 (5'- CTT GGA TGG TGA TGC ATG GCC - 3') and VAR2 (5'- ATC TTT CAA TCG GTA GGA GCG AC - 3'). The reaction mixture contained buffer 10 mM Tris-HCl with pH 8.3, 50 mM KCl, 200 μM of each of the four dNTPs, 10 μM of each oligonucleotide primer, 1 μg DNA template and 0.5 U of ampli-*Taq *DNA polymerase (Invitrogen, USA) to a final volume of 25 μL. All amplification cycles included to an initial cycle of 95°C for 15 min, followed by 30 cycles of 94°C for 1 min, 68°C for 1 min, and 72°C for 1 min, then a final extension at 72°C for 10 min.

#### *Cytochrome B *gene analyses

The *cytochrome B (Cyt B) *sequences were amplified by PCR using sets of primers: PC1 (5'- GCTACAGGTGCATCTCTTGTATTC - 3') and PC2 (5' - CACTTACAGTATATCCTCCACATAACCA - 3'). A reaction mixture of buffer 10 mM Tris-HCl, pH 8.3, 50 mM KCl, 200 μM of each of the four dNTPs, 10 μM of each oligonucleotide primer, 1 μg DNA template and 0.5 U of ampli-*Taq *DNA polymerase (Invitrogen, USA). The amplification conditions were as follows: first, 1 min at 94°C, followed by 30 cycles with 0.5 min of denaturation at 94°C, annealing at 40°C for 0.5 min and elongation at 72°C for 1.5 min. After 30 cycles, a final elongation step at 72°C for 3 min was carried out. The agarose gels were stained with ethidium bromide and analysed with a Gel Doc 2000 illuminator (Bio-Rad Laboratories, USA).

### Sequence alignment and phylogenetic analyses

The sequences were edited and aligned with the programs MEGA (version 4.1) and BioEdit Sequence Alignment Editor by the CLUSTAL W tool. Phylogenetic analyses were performed with neighbor-joining (NJ), using the program MEGA (version 4.1), with *p *distance which takes into account the possibility of high bias in the transition/transversion and in G+C content, derived of the position of the first, second and third codon [45]. The reliability of the NJ trees is assessed by the bootstrap method with 500 replications [46].

### Assessment of the serological response against *P. vivax CS *genotypes in the current infections

IgG antibodies against four CSP peptides (N-terminal [N] and C-terminal [C], repetitive region corresponding to the VK210 [R] and repetitive region corresponding to the VK247 [V]) [47], MSP-1 N- terminal fragment (r*Pv*200L) [48], recombinant peptide of the AMA-1 [49] and of the DBP [50] were detected by ELISA (enzyme-linked immunosorbent assay), in plasma samples in infected individuals with *P. vivax CS *genotypes.

### Statistical analysis

The serological data were performed using R version 2.8.1 statistical software The R Foundation for Statistical Computing, Vienna, Austria [51]. Differences among the frequencies of responders were analysed using Pearson's chi-square or, alternatively, the Fisher's exact test. Differences were considered significant when *p*-value ≤ 0.05.

## Results

### Phylogeny of *P. vivax CS *genotypes, VK210 and *P. vivax*-like

**Figure 1:**
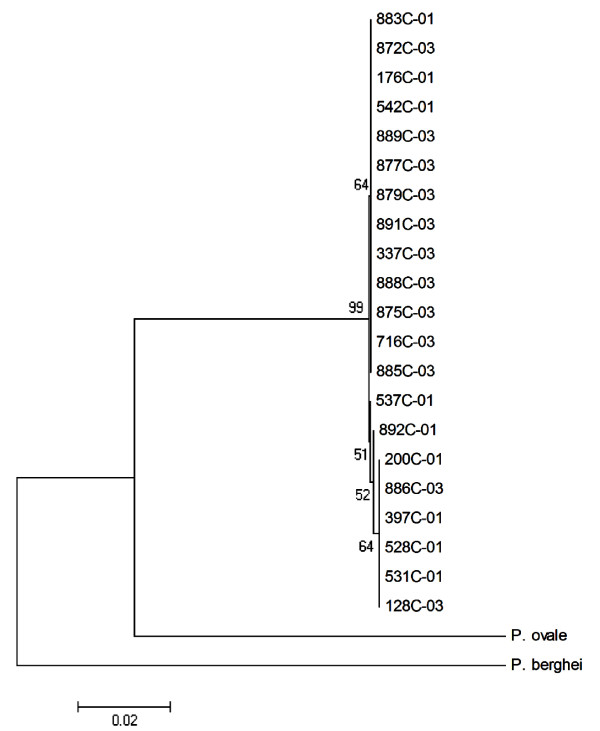
**Neighbor-joining tree of the *18 SSU rRNA *gene based in *p *distance, including transitions and transversions**. The numbers are bootstrap percent values based on 500 replications. The end 01 and 03 are corresponding of the VK210 and *P. vivax*-like genotypes, respectively.

**Figure 2:**
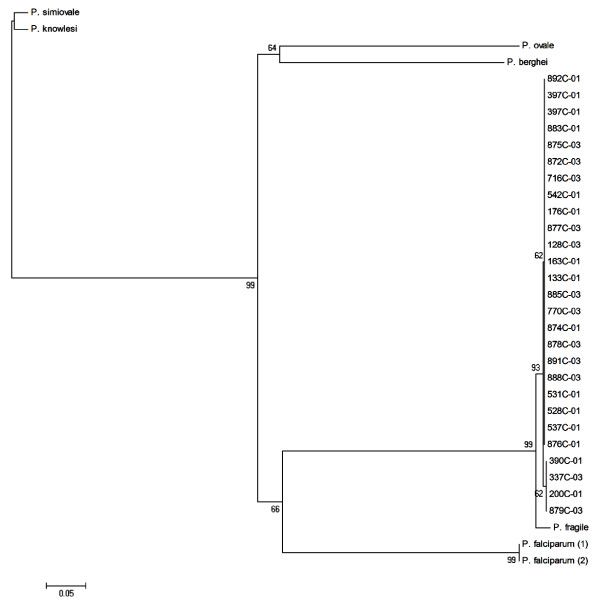
**Neighbor-joining tree of the *Cyt B *gene based in *p *distance, including transitions and transversions**. The numbers are bootstrap percent values based on 500 replications. The end 01 and 03 are corresponding of the VK210 and *P. vivax*-like genotypes, respectively.

The analyses of the two markers show high similarity among the *P. vivax CS *genotypes, with nucleotide diversity equal to zero (*p *= 0.224, t Student's test), positioning the genotypes VK210 and *P. vivax*-like in the same clade (Figure 1 and Figure 2). The genetic distances between *CS *genotypes from the *Plasmodium *species analysed are described in Additional file 2 and Additional file 3: Genetic distances between *18 SSU rRNA *and *Cyt B *genes from *Plasmodium *spp. The blood samples infected with VK247 genotypes were not included in this study, because of the reduced numbers of VK247 samples (*n *= 4) and low quality of the material.

### Antibody response against the CSP, MSP-1, AMA-1 and DBP peptides

A higher frequency IgG antibody against the N- and C-terminal regions of the *P. vivax *CSP was found as compared to the immune response to the R- and V- repetitive regions (*p *= 0.0005, Fisher's exact test). Antibody responses against the peptides of the CSP repetitive central region [R] and [V] compared displayed a lower frequency against the [V] peptide, which corresponds to the central region of the VK247 variant, in individuals with VK210 genotype (*p *< 0.005, Fisher's exact test). When *P. vivax*-like genotype was present in the infection a lower antibody response against [R] and [V] peptides was observed (*p *= 0.003, Fisher's exact test). None other significant association was found with de *CS *genotypes in the infection (Table 1).

**Table 1:** Frequency of antibody response against CSP peptides in the infections with the *P. vivax CS *genotypes.

Peptides analyzed	*CS *genotypes present in the current infection (*n*)						
	
	1 (34)	2 (4)	3 (18)	1 + 2 (12)	1 + 3 (8)	1 + 2 + 3 (4)	Total (80)**
[N] - terminal	85,3	100	77,8	75	100	75	83,7
[C] - terminal	73,5	100	88,9	100	75	100	83,7
[R] - VK 210	70,6	100	38,9*	100	87,5	75	62,5
[V] - VK 247	41,1*	75	55,6*	83,3	50	50	50

*p = 0.003, Fisher's Exact test. **p = 0.0005, Fisher's Exact test. 1: VK210; 2: VK247; 3: *P. vivax*-like.

A high frequency of antibody response against MSP-1 and AMA-1 peptides was observed for all *P. vivax CS *genotypes in comparison to the same frequency for DBP. A high frequency of antibody response against MSP-1 (r*Pv*200L) and AMA-1 peptides was observed for all the *P. vivax CS *genotypes (Table 2) in comparison to same frequency for DBP (*p *= 0.003, Fisher's exact test). However, significant differences were not observed among the immune responses of individuals infected with the *P. vivax CS *genotypes for none of the analysed peptides.

**Table 2:** Frequency of antibody response against merozoite antigens in the infections with the *P. vivax CS *genotypes.

Peptides analyzed	*CS *genotypes present in the current infection (*n*)						
	
	1 (51)	2 (4)	3 (23)	1 + 2 (14)	1 + 3 (8)	1 + 2 + 3 (4)	Total (104)
*Pv*200L	92.2	100	78.2	100	87.5	100	90.4

	1 (39)	2 (4)	3 (18)	1 + 2 (13)	1 + 3 (8)	1 + 2 + 3 (4)	Total (86)

AMA-1	92.3	75	94.4	100	87.5	75	91.9
rII-DBP	41	50	33.3	38.5	50	50	40.7*

*p = 0,003 Fisher's Exact test. 1: VK210; 2: VK247; 3: *P, vivax*-like.

## Discussion

Starting from the description of the *P. vivax CS *genotypes, VK210, VK247 and *P. vivax*-like, several studies proposed the existence of differences among those that seem to go besides variations in the repetitive portion of the protein, as geographical distribution, transmission intensity, vectorial competence, immune and treatment responses and drug resistance [11,18,19,32-34]. The real implication of the genetic variation in this region of the *P. vivax *genome has been questioned for a long time. Although previous studies of our group have observed significant association between VK210 and the Duffy blood group [17], this work presents here evidences of this variation is limited to the CSP central portion.

Studies based on molecular marker analysis represent an important tool for the phylogenetic characterization of malaria parasites. Similarities between *P. vivax*-like and *Plasmodium simiovale *have been reported in phylogenetic studies with conserved domains of the *CS *gene and, some authors suggested that this variant genotype could be a subspecies or a new species [6]. However, previous phylogenetic studies were designed with the *CS *gene as the only molecular marker in a way that prevents an explanation on the evolutionary relationship among the three *CS *genotypes as well as its relativity to other primate parasites that possess molecular similarities with *P. vivax *[35]. In this study, the results were obtained through the phylogenetic analysis of the *18 SSU rRNA *and *Cyt B Plasmodium *spp. recognized markers and surprisingly showed diversity equal to zero between both *P. vivax CS *genotypes, VK210 and *P. vivax*-like. This analyses positioned VK210 and *P. vivax*-like as members of the same clade, in accordance with previous data [35]. Although the absence of VK247 genetic sequences, a limitation of the present study, the results point to the fact that *P. vivax CS *genotypes merely represent markers of intra-specific genetic variations.

Supporting the above mentioned hypothesis, the evaluation of the serological response profile against the different parasite peptides corroborates the idea that this variation is restricted to central portion of CSP, once significant associations were not observed between the presence of certain genotype and frequency of the antibodies responses against the three analysed merozoite peptides, MSP1 (*Pv*200L), AMA-1, DBP and against the CSP conserved fractions in the sporozoite, N-terminal and C-terminal. Besides, when the antibody responses against the peptides corresponding to the CSP repetitive central region were evaluated, significant associations were detected against the peptides [R] and [V], which corresponds to the protein sequences of VK210 and VK247 genotypes, respectively. In individuals infected by the VK210 genotype, a lower antibody response against [V] was observed whereas in those infected by the *P. vivax*-like genotype observed an even lower antibody response against these two fragments ([R] and [V]) were observed. Once VK210 represents the classic *P. vivax CS *variant form and also the most prevalent in all Brazilian endemic areas [17], these results were expected. Moreover, the repeated region of the *P. vivax*-like *CS *is the most genetically distinct compared to the other variants [5,6].

The report that variations in the central repetitive portion of CSP do not provide significant differences in antibody responses against *P. vivax *merozoite and sporozoite conserved regions peptides represents key information in the future design of vaccine assays. On the other hand, studies based in CSP should consider the influences of this variation in the modulation of the epidemiology and to consider the use of chimerical constructs including the sequences of the different *CS *genotypes in order to obtain a vaccine indeed protecting.

## Conclusion

These results target that the differences among the *P. vivax CS *variants are restrict to the central repeated region of the protein, mostly nucleotide variation with important serological consequences. This variation can represent intra-specific biological signatures that must be considered for *P. vivax *CSP malaria vaccine trial.

## Competing interests

The authors declare that they have no competing interests.

## Authors' contributions

WCSN carried out the molecular genetic studies, participated in the sequence alignment, phylogenetics analyses and drafted the manuscript. LMSM carried out the immunoassays and participated in the design of the study and performed of the statistical analysis part. ISS, LHC and MGC designed serological experiments and provided reagents. GCC, VSCAC, ISS, LHC, SH, MAH, ARBR critically revised the manuscript. CMAC participated in the sequence alignment and phylogenetics analyses. RLDM conceived of the study, and participated in its design and coordination and helped to draft the manuscript. All authors read and approved the final manuscript.

## Supplementary Material

Additional file 1 **Hosts type, geographic origins and GenBank accession numbers of the out groups**. GenBank accession numbers. Click here for file

Additional file 2 **Genetic distances between *18 SSU rRNA *genes from *Plasmodium *spp**. Genetic distances. Click here for file

Additional file 3 **Genetic distances between *Cyt B *genes from *Plasmodium *spp**. Genetic distances. Click here for file
