# Supplementary material for: Plasmodium vivax circumsporozoite genotypes: a limited variation or new subspecies with major biological consequences?
Source: Malar J. 2010 Jun 23;9:178. doi: 10.1186/1475-2875-9-178 (PMC2908638; doi:10.1186/1475-2875-9-178)
Supplement: Additional file 1 — Hosts type, geographic origins and GenBank accession numbers of the out groups. GenBank accession numbers. [file 1475-2875-9-178-S1.DOC]

| **Additional file 1.** Hosts type, geographic origins and GenBank accession numbers of the out groups. | | | |
| --- | --- | --- | --- |
|  |  |  |  |
| **Parasite** | **Natural Host** | **Geographic Origin** | **Número de acesso** |
| *P. ovale* | Humans | Africa, Nigeria | L48987.1 |
| *P. berghei* | *Grammomys surdaster* | Katanga, Congo | M14599.1 |
| *P. falciparum (1)* | Humans | Thailand | M99416.1 |
| *P. falciparum (2)* | Humans | Thailand | NC_002375.1 |
| *P. fragile* | *Macaca radiat, M. sinisca* | South of India, Sri Lanka | NC_012369.1 |
| *P. berghei* | *Grammomys surdaster* | Katanga, Congo | EU254525.1 |
| *P. ovale* | Humans | Africa, Nigeria | FJ409567.1 |
| *P. knowlesi* | Old World Monkeys | Asia, Africa | NC_007232 |
| *P. simiovale* | *M. sínica* | Sri Lanka | AB434920 |
