# Supplementary material for: Plasmodium vivax circumsporozoite genotypes: a limited variation or new subspecies with major biological consequences?
Source: Malar J. 2010 Jun 23;9:178. doi: 10.1186/1475-2875-9-178 (PMC2908638; doi:10.1186/1475-2875-9-178)
Supplement: Additional file 2 — Genetic distances between 18 SSU rRNA genes from Plasmodium spp. Genetic distances. [file 1475-2875-9-178-S2.DOC]

**Additional file 2.** Genetic distances between *18 SSU RNAr* genes from *Plasmodium* spp.

[ 1 2 3 4 5 6 7 8 9 10 11 12 13 14 15 16 17 18 19 20 21 22 23 ]

[ 1]

[ 2] 0.000

[ 3] 0.000 0.003

[ 4] 0.000 0.000 0.003

[ 5] 0.000 0.003 0.000 0.003

[ 6] 0.000 0.000 0.000 0.000 0.000

[ 7] 0.000 0.000 0.003 0.000 0.003 0.000

[ 8] 0.000 0.003 0.000 0.003 0.000 0.000 0.003

[ 9] 0.000 0.003 0.000 0.003 0.000 0.000 0.003 0.000

[10] 0.000 0.000 0.003 0.000 0.003 0.000 0.000 0.003 0.003

[11] 0.000 0.003 0.000 0.003 0.000 0.000 0.003 0.000 0.000 0.003

[12] 0.000 0.000 0.003 0.000 0.003 0.000 0.000 0.003 0.003 0.000 0.003

[13] 0.000 0.000 0.003 0.000 0.003 0.000 0.000 0.003 0.003 0.000 0.003 0.000

[14] 0.000 0.000 0.003 0.000 0.003 0.000 0.000 0.003 0.003 0.000 0.003 0.000 0.000

[15] 0.000 0.000 0.003 0.000 0.003 0.000 0.000 0.003 0.003 0.000 0.003 0.000 0.000 0.000

[16] 0.000 0.000 0.003 0.000 0.003 0.000 0.000 0.003 0.003 0.000 0.003 0.000 0.000 0.000 0.000

[17] 0.000 0.000 0.003 0.000 0.003 0.000 0.000 0.003 0.003 0.000 0.003 0.000 0.000 0.000 0.000 0.000

[18] 0.000 0.000 0.003 0.000 0.003 0.000 0.000 0.003 0.003 0.000 0.003 0.000 0.000 0.000 0.000 0.000 0.000

[19] 0.000 0.000 0.003 0.000 0.003 0.000 0.000 0.003 0.003 0.000 0.003 0.000 0.000 0.000 0.000 0.000 0.000 0.000

[20] 0.000 0.000 0.003 0.000 0.003 0.000 0.000 0.003 0.003 0.000 0.003 0.000 0.000 0.000 0.000 0.000 0.000 0.000 0.000

[21] 0.000 0.003 0.000 0.003 0.000 0.000 0.003 0.000 0.000 0.003 0.000 0.003 0.003 0.003 0.003 0.003 0.003 0.003 0.003 0.003

[22] 0.183 0.183 0.183 0.183 0.183 0.183 0.183 0.183 0.183 0.183 0.183 0.183 0.183 0.183 0.183 0.183 0.183 0.183 0.183 0.183 0.183

[23] 0.129 0.131 0.134 0.131 0.134 0.132 0.131 0.134 0.134 0.131 0.134 0.131 0.131 0.131 0.131 0.131 0.131 0.131 0.131 0.131 0.134 0.212

The end 01 and 03 are corresponding of the VK210 and *P. vivax*-like genotypes, respectively. 1. 537C-01, 2. 883C-01, 3. 397C-01, 4. 542C-01, 5. 528C-01, 6. 892C-01, 7. 176C-01, 8. 531C-01, 9. 200C-01, 10. 889C-03, 11. 886C-03, 12. 888C-03, 13. 879C-03, 14. 716C-03, 15. 872C-03, 16. 337C-03, 17. 877C-03, 18. 891C-03, 19. 885C-03, 20. 875C-03, 21. 128C-03, 22. *P. berghei*, 23. *P. ovale*.
