# Supplementary material for: Plasmodium vivax circumsporozoite genotypes: a limited variation or new subspecies with major biological consequences?
Source: Malar J. 2010 Jun 23;9:178. doi: 10.1186/1475-2875-9-178 (PMC2908638; doi:10.1186/1475-2875-9-178)
Supplement: Additional file 3 — Genetic distances between Cyt B genes from Plasmodium spp. Genetic distances. [file 1475-2875-9-178-S3.DOC]

**Additional file 3.** Genetic distances between *Cyt B* genes from *Plasmodium* spp.

**[1 2 3 4 5 6 7 8 9 10 11 12 13 14 15 16 17 18 19 20 21 22 23 24 25 26 27 28 29 30 31 32 33 34 ]**

[ 1]

[ 2] 0.000

[ 3] 0.006 0.006

[ 4] 0.000 0.000 0.006

[ 5] 0.000 0.000 0.006 0.000

[ 6] 0.000 0.000 0.006 0.000 0.000

[ 7] 0.000 0.000 0.006 0.000 0.000 0.000

[ 8] 0.000 0.000 0.006 0.000 0.000 0.000 0.000

[ 9] 0.000 0.000 0.006 0.000 0.000 0.000 0.000 0.000

[10] 0.000 0.000 0.006 0.000 0.000 0.000 0.000 0.000 0.000

[11] 0.000 0.000 0.006 0.000 0.000 0.000 0.000 0.000 0.000 0.000

[12] 0.000 0.000 0.006 0.000 0.000 0.000 0.000 0.000 0.000 0.000 0.000

[13] 0.000 0.000 0.006 0.000 0.000 0.000 0.000 0.000 0.000 0.000 0.000 0.000

[14] 0.000 0.000 0.006 0.000 0.000 0.000 0.000 0.000 0.000 0.000 0.000 0.000 0.000

[15] 0.000 0.000 0.006 0.000 0.000 0.000 0.000 0.000 0.000 0.000 0.000 0.000 0.000 0.000

[16] 0.000 0.000 0.006 0.000 0.000 0.000 0.000 0.000 0.000 0.000 0.000 0.000 0.000 0.000 0.000

[17] 0.000 0.000 0.006 0.000 0.000 0.000 0.000 0.000 0.000 0.000 0.000 0.000 0.000 0.000 0.000 0.000

[18] 0.000 0.000 0.006 0.000 0.000 0.000 0.000 0.000 0.000 0.000 0.000 0.000 0.000 0.000 0.000 0.000 0.000

[19] 0.006 0.006 0.000 0.006 0.006 0.006 0.006 0.006 0.006 0.006 0.006 0.006 0.006 0.006 0.006 0.006 0.006 0.006

[20] 0.000 0.000 0.006 0.000 0.000 0.000 0.000 0.000 0.000 0.000 0.000 0.000 0.000 0.000 0.000 0.000 0.000 0.000 0.006

[21] 0.000 0.000 0.006 0.000 0.000 0.000 0.000 0.000 0.000 0.000 0.000 0.000 0.000 0.000 0.000 0.000 0.000 0.000 0.006 0.000

[22] 0.000 0.000 0.006 0.000 0.000 0.000 0.000 0.000 0.000 0.000 0.000 0.000 0.000 0.000 0.000 0.000 0.000 0.000 0.006 0.000 0.000

[23] 0.000 0.000 0.006 0.000 0.000 0.000 0.000 0.000 0.000 0.000 0.000 0.000 0.000 0.000 0.000 0.000 0.000 0.000 0.006 0.000 0.000 0.000

[24] 0.006 0.006 0.000 0.006 0.006 0.006 0.006 0.006 0.006 0.006 0.006 0.006 0.006 0.006 0.006 0.006 0.006 0.006 0.000 0.006 0.006 0.006 0.006

[25] 0.000 0.000 0.006 0.000 0.000 0.000 0.000 0.000 0.000 0.000 0.000 0.000 0.000 0.000 0.000 0.000 0.000 0.000 0.006 0.000 0.000 0.000 0.000 0.006

[26] 0.006 0.006 0.000 0.006 0.006 0.006 0.006 0.006 0.006 0.006 0.006 0.006 0.006 0.006 0.006 0.006 0.006 0.006 0.000 0.006 0.006 0.006 0.006 0.000 0.006

[27] 0.000 0.000 0.006 0.000 0.000 0.000 0.000 0.000 0.000 0.000 0.000 0.000 0.000 0.000 0.000 0.000 0.000 0.000 0.006 0.000 0.000 0.000 0.000 0.006 0.000 0.006

[28] 0.634 0.634 0.626 0.634 0.632 0.634 0.632 0.634 0.632 0.632 0.632 0.632 0.632 0.632 0.632 0.632 0.634 0.634 0.626 0.632 0.634 0.634 0.634 0.626 0.634 0.626 0.632

[29] 0.029 0.029 0.034 0.029 0.029 0.029 0.029 0.029 0.029 0.029 0.029 0.029 0.029 0.029 0.029 0.029 0.029 0.029 0.034 0.029 0.029 0.029 0.029 0.034 0.029 0.034 0.029 0.644

[30] 0.634 0.634 0.626 0.634 0.632 0.634 0.632 0.634 0.632 0.632 0.632 0.632 0.632 0.632 0.632 0.632 0.634 0.634 0.626 0.632 0.634 0.634 0.634 0.626 0.634 0.626 0.632 0.000 0.644

[31] 0.697 0.697 0.701 0.697 0.695 0.697 0.695 0.697 0.695 0.695 0.695 0.695 0.695 0.695 0.695 0.695 0.697 0.697 0.701 0.695 0.697 0.697 0.697 0.701 0.697 0.701 0.701 0.623 0.701 0.623

[32] 0.697 0.697 0.701 0.697 0.695 0.697 0.695 0.697 0.695 0.695 0.695 0.695 0.695 0.695 0.695 0.695 0.697 0.697 0.701 0.695 0.697 0.697 0.697 0.701 0.697 0.701 0.701 0.646 0.701 0.646 0.669

[33] 0.697 0.697 0.701 0.697 0.695 0.697 0.695 0.697 0.695 0.695 0.695 0.695 0.695 0.695 0.695 0.695 0.697 0.697 0.701 0.695 0.697 0.697 0.697 0.701 0.697 0.701 0.701 0.651 0.701 0.651 0.663 0.034

[34] 0.674 0.674 0.678 0.674 0.672 0.674 0.672 0.674 0.672 0.672 0.672 0.672 0.672 0.672 0.672 0.672 0.674 0.674 0.678 0.672 0.674 0.674 0.674 0.678 0.674 0.678 0.678 0.714 0.672 0.714 0.589 0.646 0.640

The end 01 and 03 are corresponding of the VK210 and *P. vivax*-like genotypes, respectively. 1. 872C-03, 2. 892C-01, 3. 200C-01, 4. 875C-03, 5. 874C-01, 6. 176C-01, 7. 885C-03, 8. 877C-03, 9. 888C-03, 10. 531C-01, 11. 528C-01, 12. 891C-03, 13. 878C-03, 14. 133C-01, 15. 770C-03, 16. 876C-01, 17. 163C-01, 18. 128C-03, 19. 390C-01, 20. 537C-01, 21. 397C-01, 22. 716C-03, 23. 883C-01, 24. 337C-03, 25. 542C-01, 26. 879C-03, 27. 397C-01, 28. *P. falciparum* (1), 29. *P. fragile*, 30. *P. falciparum* (2), 31. *P. ovale*, 32. *P. knowlesi*, 33. *P. simiovale*, 34. *P. berghei*.
